# Diabetic kidney disease: from pathogenesis to multimodal therapy–current evidence and future directions

**DOI:** 10.3389/fmed.2025.1631053

**Published:** 2025-08-08

**Authors:** Hui Zhang, Keding Wang, Hairui Zhao, Bowen Qin, Xiaojing Cai, Manyi Wu, Junhua Li, Jielian Wang

**Affiliations:** ^1^Department of Nephrology, Tianyou Hospital, Wuhan University of Science and Technology, Wuhan, China; ^2^Department of Nephrology, Tongii Hospital, Tongji Medical College, Huazhong University of Science and Technology, Wuhan, China

**Keywords:** diabetic kidney disease (DKD), sodium-glucose co-transporter 2 inhibitors (SGLT2i), finerenone, gut-kidney axis, cardiorenal protection

## Abstract

Diabetic kidney disease (DKD) has emerged as the leading cause of chronic kidney disease (CKD) worldwide, surpassing primary glomerular disorders in prevalence. Despite recent therapeutic advances, current treatment strategies primarily alleviate symptoms rather than address the underlying pathogenic mechanisms, highlighting an urgent need for targeted, mechanism-based interventions. The pathogenesis of DKD involves a complex interplay of metabolic, hemodynamic, inflammatory, oxidative, and fibrotic pathways. Chronic hyperglycemia initiates a cascade of molecular events—including the accumulation of advanced glycation end products (AGEs), activation of the polyol pathway, enhanced protein kinase C (PKC) signaling, and mitochondrial dysfunction—culminating in glomerular hyperfiltration, podocyte injury, and progressive glomerular and tubulointerstitial fibrosis. In addition to these classical mechanisms, emerging processes such as ferroptosis (iron-dependent cell death), impaired autophagy, gut microbiota dysbiosis, and epigenetic alterations offer promising therapeutic targets. Current DKD management integrates lifestyle modifications with four cornerstone pharmacologic classes: renin–angiotensin–aldosterone system inhibitors (RAASi), sodium–glucose co-transporter 2 inhibitors (SGLT2i), glucagon-like peptide-1 receptor agonists (GLP-1 RAs), and mineralocorticoid receptor antagonists (MRAs). Notably, evidence from clinical trials suggests that simultaneous modulation of multiple pathogenic pathways provides superior cardiorenal protection compared to monotherapy. Investigational therapies—including endothelin receptor antagonists (ERAs), nuclear factor erythroid 2–related factor 2 (Nrf2) activators, and gut microbiota modulators—are under active evaluation. Additionally, Traditional Chinese Medicine (TCM) formulations have demonstrated albuminuria-lowering effects in clinical studies. Future research should prioritize biomarker-driven precision medicine approaches, enabling individualized therapy selection and development of agents that concurrently target ferroptosis and inflammation. Given the multifaceted pathophysiology of DKD, optimal management will require multimodal, patient-tailored regimens that address hyperglycemia, hypertension, inflammation, and fibrosis to effectively slow or halt disease progression.

## 1 Epidemiological background

Diabetic kidney disease (DKD) is one of the most prevalent and severe chronic microvascular complications of diabetes (1). According to the International Diabetes Federation, ~425 million adults aged 20–79 years are living with diabetes globally, reflecting a prevalence of 8.8% (2). China bears the highest disease burden, accounting for 114 million individuals—26.7% of the global diabetic population—with projections rising to 120 million by 2045 (2). DKD develops in ~20%−40% of patients with diabetes, with incidence influenced by factors such as diabetes duration, glycemic control, and genetic susceptibility (3). It has now overtaken primary glomerular diseases as the leading cause of chronic kidney disease (CKD) worldwide and substantially increases both cardiovascular and all-cause mortality risk among individuals with type 2 diabetes (T2D) (4). To address this escalating health crisis, current DKD management emphasizes four core pharmacologic classes: renin–angiotensin–aldosterone system inhibitors (RAASi), mineralocorticoid receptor antagonists (MRAs), sodium–glucose co-transporter 2 inhibitors (SGLT2i), and glucagon-like peptide-1 (GLP-1) receptor agonists (GLP-1 RAs) (5). Robust clinical evidence supports the use of combination therapy with these agents, demonstrating significant benefits for both renal preservation and cardiovascular protection. Ongoing research into targeted therapeutics and precision medicine strategies holds promise for optimizing treatment outcomes and transforming the long-term prognosis of patients with DKD (6).

## 2 Pathogenesis of DKD

DKD develops through a constellation of interconnected pathological processes—including metabolic dysregulation, hemodynamic alterations, inflammation, and fibrotic remodeling—that collectively drive progressive renal dysfunction and structural deterioration. A comprehensive understanding of these mechanisms is essential for developing effective, targeted therapies. Chronic hyperglycemia is the central initiating factor in DKD pathogenesis, activating multiple overlapping molecular cascades that cause cellular injury (7). Metabolically, persistent hyperglycemia upregulates glucose transporter-1 (GLUT1), reinforcing a self-perpetuating TGF-β1–GLUT1 signaling loop in mesangial cells that promotes abnormal extracellular matrix (ECM) accumulation, primarily of collagen and fibronectin (8). Concurrently, non-enzymatic glycation of proteins and lipids leads to the formation of advanced glycation end products (AGEs), which drive kidney injury through both RAGE-dependent and RAGE-independent pathways (9). RAGE engagement activates NF-κB signaling, leading to the release of pro-inflammatory cytokines and the generation of reactive oxygen species (ROS), while RAGE-independent pathways contribute to ECM expansion and tissue fibrosis (9–11). Hyperglycemia also triggers alternative metabolic pathways—including the polyol pathway, hexosamine biosynthesis, and protein kinase C (PKC) activation—all of which exacerbate oxidative stress and amplify transforming growth factor-beta (TGF-β)/Smad signaling (12–16). These events contribute to glomerular hypertrophy, basement membrane thickening, and mesangial expansion (17). At the cellular level, mitochondrial dysfunction and ferroptosis—an iron-dependent form of regulated cell death—initiate a self-reinforcing cycle of ROS overproduction, lipid peroxidation, and iron accumulation (18–20). Impaired autophagy and dysregulated endoplasmic reticulum (ER) stress responses further hinder the clearance of damaged cellular components, accelerating renal cell injury (21, 22). Hemodynamic abnormalities represent another critical contributor to DKD progression. Afferent arteriolar dilation, together with renin–angiotensin system (RAS) hyperactivation, results in glomerular hyperfiltration and increased intraglomerular pressure (23). This mechanical stress damages the filtration barrier and activates inflammatory pathways involving cytokines, adhesion molecules, and selectins, culminating in endothelial dysfunction, podocyte injury, mesangial cell activation, and fibrotic changes that collectively drive albuminuria and decline in renal function (8, 24, 25). In summary, sustained hyperglycemia orchestrates a multifactorial pathophysiological network involving metabolic injury, oxidative stress, hemodynamic strain, and inflammatory amplification. Effective therapeutic strategies must, therefore adopt an integrated approach, simultaneously targeting these pathogenic axes to meaningfully slow or reverse DKD progression.

Accumulating evidence highlights inflammation as a central driver in both the initiation and progression of DKD (26). Persistent hyperglycemia activates a network of interrelated inflammatory pathways that contribute to sustained renal injury. One of the principal mechanisms involves hyperglycemia-induced oxidative stress and the consequent formation of advanced glycation end products (AGEs), which in turn activate two key inflammatory cascades: the Toll-like receptor (TLR) 2/4–MyD88–NF-κB pathway and the thioredoxin-interacting protein–NOD-like receptor pyrin domain-containing 3 (NLRP3) inflammasome complex (27–29). Activation of these signaling pathways promotes the release of pro-inflammatory cytokines, including interleukin (IL)-6, IL-1β, and tumor necrosis factor-alpha (TNF-α), which compromise the integrity of the endothelial glycocalyx, increase glomerular capillary permeability, and promote microalbuminuria—an early hallmark of diabetic renal injury (26, 30–32). Inflammatory signaling further induces the expression of adhesion molecules such as vascular cell adhesion molecule-1, intercellular adhesion molecule-1, and P-selectin, facilitating the recruitment of monocytes, macrophages, and T lymphocytes into renal tissue (32). This fosters a self-reinforcing loop among infiltrating immune cells, inflammatory mediators, and adhesion molecules, perpetuating tissue damage (33). AGE-RAGE interactions intensify oxidative stress via both receptor-dependent and -independent mechanisms, while also amplifying NF-κB activity to further exacerbate the inflammatory milieu (34, 35). The heightened oxidative burden triggers gasdermin D-mediated pyroptosis and promotes partial epithelial–mesenchymal transition, contributing to glomerulosclerosis and tubulointerstitial fibrosis (36, 37). The inflammatory landscape in DKD is, thus characterized by simultaneous activation of multiple signaling cascades, dynamic immune–renal cell crosstalk, and self-amplifying feedback loops. This complex pathophysiology provides a compelling rationale for the development of multi-targeted anti-inflammatory therapies, including NLRP3 inflammasome inhibitors, NF-κB pathway modulators, and biologic agents targeting IL-6, IL-1β, and TNF-α–each showing promising potential to attenuate disease progression.

**Figure 1:**
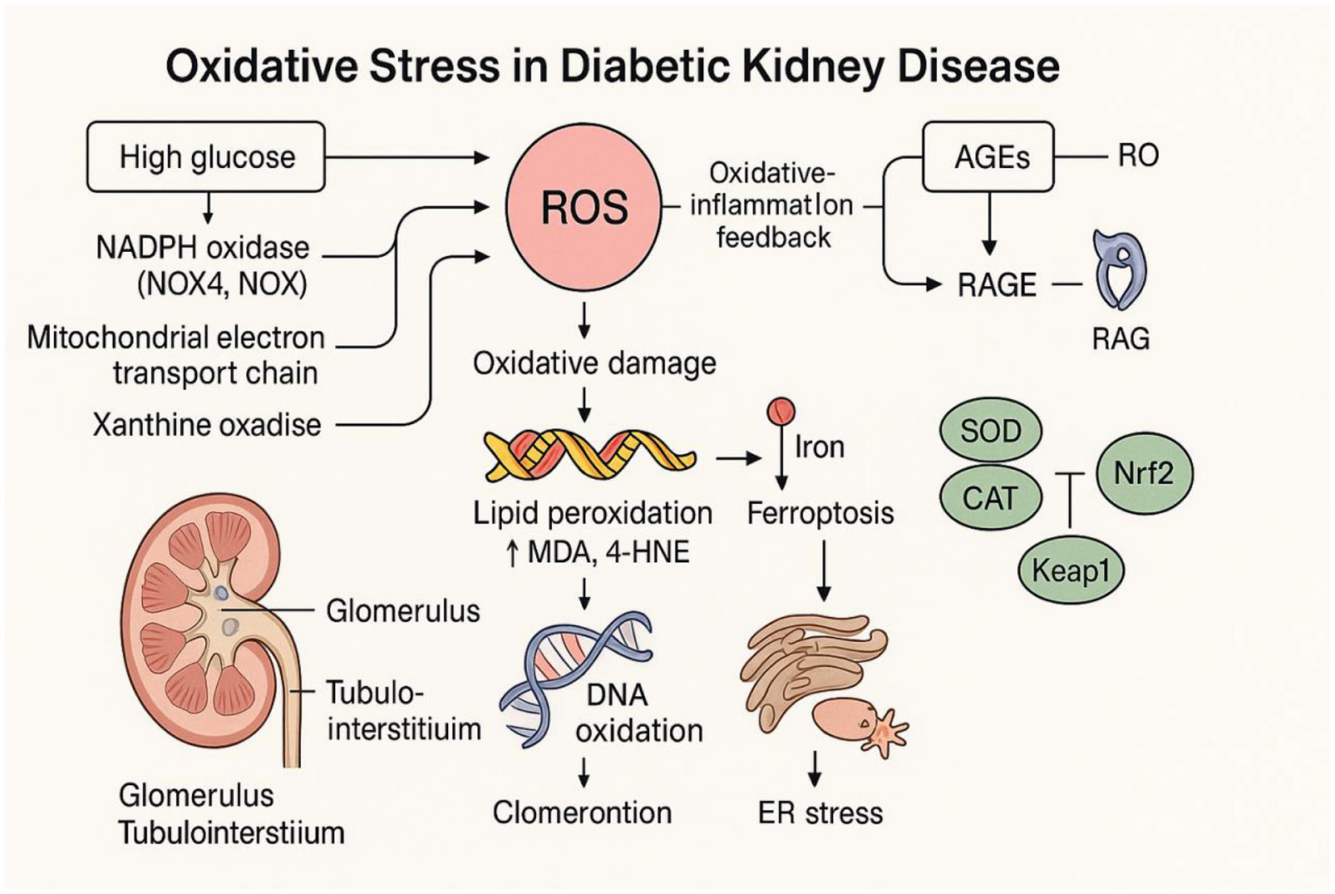
Oxidative stress pathways in DKD. This figure depicts the role of oxidative stress in DKD pathogenesis. Hyperglycemia induces excessive ROS production via NADPH oxidase (NOX4/5), mitochondrial dysfunction, and xanthine oxidase. ROS promotes lipid peroxidation, DNA damage, ferroptosis, and ER stress. AGE–RAGE signaling amplifies ROS, while Nrf2–Keap1 pathway and antioxidant enzymes (SOD, CAT, and GSH-Px) are inhibited, exacerbating oxidative damage in glomerular and tubulointerstitial compartments. ROS, reactive oxygen species; AGEs, advanced glycation end-products; RAGE, receptor for AGEs; NOX, NADPH oxidase isoforms; MDA, malondialdehyde; 4-HNE, 4-hydroxynonenal; 8-OHdG Kelch-like ECH, 8-hydroxy-2′-deoxyguanosine; SOD, superoxide dismutase; CAT, catalase; GSH-Px, glutathione peroxidase; Nrf2, nuclear factor erythroid 2–related factor 2; Keap1, Kelch-like ECH-associated protein 1; ER, endoplasmic reticulum.

Oxidative stress is a central pathological mechanism in DKD, closely intertwined with chronic hyperglycemia (35, 38). This pro-oxidative state, characterized by excessive reactive oxygen species (ROS) generation, arises through several converging pathways. Hyperglycemia enhances ROS production via hyperactivation of NADPH oxidases—particularly the NOX4 and NOX5 isoforms—dysfunction of the mitochondrial electron transport chain leading to electron leakage, and increased xanthine oxidase activity (39–41). These ROS, in turn, amplify AGE–receptor for AGE (RAGE) signaling, establishing a vicious cycle wherein oxidative damage and inflammation reinforce one another, exacerbating renal injury (35). Excess ROS inflict widespread molecular damage: lipid peroxidation yields cytotoxic byproducts such as malondialdehyde (MDA) and 4-hydroxynonenal (4-HNE); DNA oxidative lesions manifest as elevated levels of 8-hydroxydeoxyguanosine (8-OHdG); and proteins undergo oxidative modifications that impair their function (39–41). These molecular insults compromise the integrity of the glomerular filtration barrier and accelerate tubular and interstitial injury (42). Concurrently, DKD is marked by impaired endogenous antioxidant defenses. The nuclear factor erythroid 2–related factor 2 (Nrf2)–Kelch-like ECH-associated protein 1 (Keap1) axis, a key regulator of cellular redox homeostasis, exhibits diminished activity (43). Levels and activity of major antioxidant enzymes—superoxide dismutase (SOD), catalase (CAT), and glutathione peroxidase (GSH-Px)—are also significantly reduced, weakening the kidney's capacity to neutralize ROS (44, 45). Emerging evidence has identified additional oxidative mechanisms contributing to DKD progression. Ferroptosis, an iron-dependent form of regulated cell death, has been shown to play a significant role in renal injury (46). Moreover, the interplay between endoplasmic reticulum (ER) stress and oxidative stress further amplifies cellular dysfunction and promotes ROS-driven fibrogenesis (18). Together, these oxidative pathways underpin the transition from early-stage albuminuria to advanced renal failure. These insights underscore the therapeutic potential of strategies that target multiple aspects of oxidative stress in DKD. Such approaches may include inhibition of ROS-generating pathways, restoration of antioxidant enzyme function, and modulation of ferroptotic cell death—each offering promise for preserving renal function by mitigating ongoing oxidative injury. The oxidative stress pathways implicated in DKD pathogenesis are illustrated in Figure 1.

Hemodynamic dysregulation is a fundamental pathological driver of DKD, acting synergistically with metabolic disturbances to accelerate disease progression (44, 45). In the early stages of DKD, hyperglycemia disrupts normal renal hemodynamic regulation through several interrelated mechanisms. Elevated glucose levels impair tubuloglomerular feedback and promote afferent arteriolar vasodilation, resulting in glomerular hyperfiltration (47). Simultaneously, activation of the intrarenal renin–angiotensin system (RAS) induces efferent arteriolar vasoconstriction (48). These vascular alterations collectively elevate intraglomerular pressure, imposing sustained mechanical stress on the glomerular filtration apparatus (49). This persistent hemodynamic strain initiates a cascade of structural damage. Initially, mechanical forces disrupt glomerular endothelial cells and their protective glycocalyx, followed by activation of podocyte stress responses and mesangial cell proliferation with excessive extracellular matrix deposition (50, 51). These changes compromise the integrity of the glomerular filtration barrier, increasing permeability and facilitating the onset and progression of albuminuria (52, 53). Hemodynamic dysregulation is closely intertwined with inflammatory processes, forming a self-reinforcing pathological cycle (54). Local elevations in angiotensin II, imbalanced production of vasoactive mediators such as nitric oxide and prostaglandins, and Toll-like receptor 4 (TLR4)-mediated inflammatory signaling further disrupt vascular tone regulation (55–57). The resulting interplay among hyperfiltration, elevated intraglomerular pressure, and endothelial dysfunction perpetuates renal injury and accelerates functional decline (58–60). Clinical studies have identified progressive increases in the afferent-to-efferent arteriolar resistance ratio as a predictive marker for progression to end-stage kidney disease (ESKD) (49). These mechanistic insights have informed several key therapeutic developments. SGLT2i restore tubuloglomerular feedback and reduce intraglomerular pressure (61); angiotensin-converting enzyme (ACE) inhibitors and angiotensin receptor blockers (ARBs) interrupt the RAS cascade (62); while MRAs confer additional hemodynamic and anti-inflammatory benefits (63). Through distinct but complementary mechanisms, these agents improve renal hemodynamics and delay DKD progression. Therefore, precise characterization of hemodynamic abnormalities and implementation of multi-targeted therapeutic strategies are essential for interrupting disease progression and preserving long-term kidney function in patients with DKD.

Epigenetic regulation plays a pivotal role in the pathogenesis of DKD, providing a molecular basis for the phenomenon of metabolic memory—whereby transient episodes of hyperglycemia induce long-lasting pathological changes in renal cells (64, 65). Alterations in DNA methylation represent a hallmark of this epigenetic remodeling (66). DNA methyltransferase 1-mediated modifications exhibit bidirectional effects: hypermethylation of promoter regions in protective genes, such as ACTN4 (encoding α-actinin-4), suppresses their expression, while hypomethylation of pro-pathogenic genes, including matrix metalloproteinase-9, enhances their transcription (67, 68). These methylation shifts reprogram the expression of genes involved in glomerular barrier integrity and fibrogenesis (69). Moreover, dysfunction of the ten-eleven translocation enzyme–α-ketoglutarate axis impairs active DNA demethylation, stabilizing disease-associated methylation patterns (70, 71). Histone modifications are also dysregulated in DKD. Increased acetylation of histones H3 and H4 promotes transcription of genes associated with inflammation and TGF-β signaling (72). In parallel, SET7-mediated monomethylation at histone H3 lysine 4 (H3K4me1) sustains the expression of profibrotic genes such as MAP4K3 (73). Bromodomain-containing protein 4 binds acetylated histones and, together with the Ino80 chromatin remodeling complex, maintains open chromatin architecture to support persistent expression of disease-promoting gene programs (74). Non-coding RNAs represent a third major epigenetic regulatory axis in DKD. MicroRNAs (miRNAs; e.g., miR-27a, miR-93-5p), long non-coding RNAs (e.g., XIST, LINC01619), and circular RNAs (e.g., circCOL1A2, circADAM9) participate in intricate post-transcriptional regulatory networks (75–80). These non-coding RNAs modulate key processes—including endoplasmic reticulum stress, NF-κB activation, and ferroptosis—through competitive endogenous RNA interactions and cross-regulatory feedback (81–83). These multilayered epigenetic alterations help explain the persistence of renal injury despite glycemic improvement and may also contribute to transgenerational transmission of disease susceptibility. Accordingly, epigenetic-targeted therapies are under active investigation, including histone deacetylase inhibitors, DNA methyltransferase inhibitors, bromodomain and extraterminal domain protein blockers, and SIRT1 activators. These agents offer novel strategies to disrupt metabolic memory and attenuate DKD progression.

### 2.1 Gut microbiota dysbiosis in diabetic kidney disease

The intestinal microbiome plays a critical role in maintaining kidney homeostasis through complex bidirectional interactions within the gut–kidney axis (84, 85). Emerging evidence implicates gut microbial dysbiosis as an active contributor to the pathophysiology of DKD. Characteristic alterations in gut microbial composition in DKD include a marked reduction in short-chain fatty acid (SCFA)-producing bacteria—particularly butyrate-producing members of the Lachnospiraceae family and genera such as Blautia and Faecalibacterium—alongside an overgrowth of potentially pathogenic taxa, including Enterobacteriaceae and other gamma-proteobacteria (86). Mounting evidence indicates that patients with DKD commonly exhibit gut microbiota dysbiosis, altered bile acid pool composition, and aberrant expression of bile acid receptors—particularly Farnesoid X Receptor (FXR) and Takeda G protein–coupled receptor 5. These disruptions may exacerbate renal injury by activating pro-inflammatory signaling cascades, notably the NF-κB pathway and NLRP3 inflammasome, thereby enhancing cytokine production (87). This microbial imbalance contributes to disease progression through several mechanisms. Aberrant protein fermentation enhances the generation of nephrotoxic metabolites, such as indoxyl sulfate, p-cresol sulfate, and trimethylamine N-oxide (TMAO) (88–90). As renal function declines, these uremic toxins accumulate and exacerbate kidney injury by promoting oxidative stress, disrupting cellular metabolism, and activating inflammatory signaling pathways (90–92). Intestinal barrier dysfunction further amplifies renal damage. During DKD progression, hyperglycemia and microbial dysbiosis disrupt epithelial tight junctions, increasing gut permeability and enabling translocation of endotoxins—particularly lipopolysaccharide (LPS)—into the systemic circulation (93, 94). LPS activates the Toll-like receptor 4 (TLR4)/NF-κB pathway, sustaining systemic inflammation and accelerating renal decline (95). A reciprocal relationship exists between kidney dysfunction and gut microbial alterations: renal impairment, influenced by dietary restrictions, antibiotic exposure, slowed gastrointestinal transit, altered luminal pH, and urea accumulation, reshapes the intestinal environment, further promoting dysbiosis and perpetuating kidney injury (96). While DKD shares many features of gut dysbiosis observed in other forms of CKD, it exhibits unique microbial patterns driven by diabetes-specific metabolic disturbances (92, 97). Compared with non-diabetic kidney disease, DKD is associated with more profound depletion of SCFA-producing bacteria, resulting in reduced levels of beneficial microbial metabolites such as butyrate, propionate, and acetate (98). These SCFAs exert anti-inflammatory effects via activation of G-protein–coupled receptors and inhibition of histone deacetylases; their deficiency aggravates renal inflammation and fibrosis (99). Additionally, DKD is characterized by aberrant amino acid metabolism. Enhanced microbial degradation of tryptophan and phenylalanine increases production of nephrotoxic derivatives—such as phenyl sulfate and indole compounds—which contribute to fibrosis through oxidative stress, mitochondrial dysfunction, and NLRP3 inflammasome activation (100–102). Hyperglycemia itself directly alters the gut microbiota, favoring expansion of pathogenic species while suppressing beneficial taxa (103, 104). AGEs further modify microbial metabolic outputs and promote uremic toxin production (105). Diabetes-related gastrointestinal complications, including gastroparesis and altered motility, contribute to small intestinal bacterial overgrowth, exacerbating microbial imbalance (106). Elevated TMAO levels in DKD also downregulate tight junction proteins, increasing intestinal permeability (107, 108). This “leaky gut” phenotype—driven by hyperglycemia, uremia, and microbial toxins—establishes a destructive cycle of barrier dysfunction in both the intestine and kidney. Given the central role of gut–kidney interactions in DKD pathogenesis, microbiota-targeted interventions—including probiotics, prebiotics, synbiotics, and fecal microbiota transplantation—represent promising therapeutic strategies. A more refined understanding of diabetes-specific dysbiosis may facilitate the development of precision microbiota-based interventions for DKD prevention and treatment.

### 2.2 Exosome signaling in DKD

Exosomes—first characterized in 1982—are membrane-bound extracellular vesicles that have emerged as pivotal mediators of intercellular communication and key contributors to disease pathogenesis. In DKD, recent evidence indicates that hyperglycemia and albuminuria stimulate increased exosome production from glomerular endothelial cells and tubular epithelial cells (109–111). These vesicles actively propagate proinflammatory and profibrotic signals within the renal microenvironment (109, 112). Under hyperglycemic conditions, mesangial cell–derived exosomes have been shown to upregulate transforming growth factor-beta (TGF-β) expression and activate the TGF-β1/PI3K–Akt signaling pathway, ultimately promoting podocyte injury and fibrotic remodeling (113). Additionally, exosomes serve as carriers of microRNAs (miRNAs) that modulate mesangial cell proliferation and interstitial fibrosis—two key pathological features of progressive DKD (114–116). These findings underscore the role of exosomes not merely as passive biomarkers, but as active participants in the molecular signaling networks that drive diabetic kidney injury.

### 2.3 Autophagy dysfunction in DKD

Autophagy is a fundamental cellular process responsible for the degradation and recycling of damaged organelles and misfolded proteins, playing a vital role in maintaining renal homeostasis. In DKD, impaired autophagy has been identified as a central pathogenic mechanism contributing to podocyte injury, albuminuria, and tubulointerstitial damage (117). Experimental studies demonstrate that enhancing autophagic flux ameliorates structural and functional renal injury, reduces proteinuria, and delays disease progression (118–120). These findings support the concept that autophagy dysregulation is involved throughout the course of DKD—from early glomerular changes to advanced stages of kidney failure. Given its essential role in cellular quality control, the autophagy pathway represents a promising therapeutic target (121). Interventions aimed at restoring autophagic function may provide a novel approach to disrupt the pathological cascade underlying DKD, offering potential to slow or halt the progression of renal injury in diabetic patients.

### 2.4 Ferroptosis activation in DKD

Ferroptosis is a regulated, iron-dependent form of cell death distinct from apoptosis, necrosis, and autophagy. Characterized by excessive lipid peroxidation and impaired antioxidant defenses, ferroptosis has been increasingly implicated in the pathogenesis of DKD (122–124). Experimental evidence from diabetic animal models and human kidney tubular epithelial cells exposed to hyperglycemic conditions demonstrates hallmark features of ferroptosis, including mitochondrial shrinkage and disrupted redox homeostasis (122, 125, 126). Inhibition of ferroptosis has been shown to confer renoprotective effects and attenuate DKD progression (122). Renal biopsy specimens from DKD patients reveal elevated expression of ferroptosis-associated proteins—such as acyl-CoA synthetase long-chain family member 4 (ACSL4) and prostaglandin-endoperoxide synthase 2—accompanied by marked downregulation of glutathione peroxidase 4 (GPX4), a key suppressor of ferroptosis (127). These molecular signatures support the contribution of ferroptotic cell death to DKD pathogenesis and identify ferroptosis as a promising therapeutic target. Interventions aimed at restoring redox balance, chelating iron, or directly inhibiting ferroptosis-related enzymes may offer novel strategies for renal protection in diabetic patients.

### 2.5 Epigenetic modifications in DKD

Epigenetic regulation—including DNA methylation, histone modifications, and non-coding RNA-mediated gene silencing—plays a crucial role in the initiation and progression of DKD (128). These heritable yet reversible modifications influence gene expression without altering the underlying DNA sequence, thereby modulating cellular responses to hyperglycemia and inflammatory stimuli (129, 130). Among epigenetic mechanisms, histone methylation has garnered particular attention for its role in shaping chromatin structure and regulating transcription of key profibrotic genes such as transforming growth factor-beta 1 (TGF-β1) and type I collagen (131, 132). Aberrant histone methylation patterns have been linked to persistent inflammation, fibrogenesis, and activation of programmed cell death pathways in renal tissues, contributing to progressive loss of kidney function in DKD (133). Importantly, the reversible nature of epigenetic modifications renders them attractive targets for therapeutic intervention. Pharmacological modulation of histone methyltransferases, DNA methyltransferases, and chromatin-binding proteins may enable reprogramming of disease-associated gene expression patterns (134). As such, targeting epigenetic dysregulation represents a promising strategy to halt or potentially reverse DKD progression in clinical settings.

## 3 Holistic approaches for managing DKD

The complex and multifactorial pathophysiology of DKD necessitates an integrated therapeutic approach that targets multiple pathogenic pathways concurrently. This section outlines a structured framework for DKD management, incorporating glycemic control, blood pressure regulation, lipid management, lifestyle modifications, evidence-based pharmacologic therapies, and coordinated multidisciplinary care.

### 3.1 Glycemic control strategies in DKD

Achieving and maintaining optimal glycemic control is fundamental to both the prevention and progression attenuation of DKD. The ADVANCE trial demonstrated that reducing hemoglobin A1c (HbA1c) levels below 6.5% was associated with a 21% reduction in nephropathy risk and a 30% reduction in proteinuria (135, 136). Similarly, in type 1 diabetes, the Diabetes Control and Complications Trial (DCCT) established that intensive glucose control reduced the risk of DKD progression by ~60% (137). These findings are supported by meta-analyses of multiple clinical trials, which show significant reductions in the incidence of both microalbuminuria (relative risk 0.86) and macroalbuminuria (relative risk 0.74) with improved glycemic management (138). However, individualized treatment remains essential. Patients with advanced kidney dysfunction may derive limited renal benefit from intensive glucose lowering and are at heightened risk of hypoglycemia (139). Recent studies also suggest that glycemic variability—fluctuations in glucose levels—may be as important as mean glycemia in predicting adverse renal outcomes. Thus, therapeutic strategies should balance glycemic targets with safety considerations, particularly in patients with reduced renal reserve (140).

### 3.2 Hypertension management in DKD patients

Blood pressure regulation is a cornerstone of DKD management. Current Kidney Disease: Improving Global Outcomes (KDIGO) guidelines recommend maintaining blood pressure below 130/80 mmHg, with more stringent targets advised for individuals with albuminuria, due to their heightened risk of renal and cardiovascular events (141, 142). Evidence from the UK Prospective Diabetes Study (UKPDS) demonstrated that effective blood pressure control significantly reduces both microvascular and macrovascular complications in patients with diabetes (143). Among antihypertensive agents, inhibitors of the renin–angiotensin–aldosterone system (RAAS)—particularly Cs (ACEIs)—have shown superior renal benefits. A subanalysis of the African American Study of Kidney Disease and Hypertension (AASK) highlighted ramipril's efficacy, demonstrating a 22%−38% reduction in composite renal endpoints compared to other antihypertensive agents (144). These findings have firmly established RAAS blockade as the foundational antihypertensive strategy in DKD. Selection of additional agents—such as calcium channel blockers or diuretics—should be guided by comorbid conditions, volume status, and patient-specific factors to achieve individualized and sustained blood pressure targets (145, 146).

### 3.3 Managing abnormal lipid profiles in DKD

Dyslipidemia is a significant modifiable risk factor for cardiovascular disease in patients with DKD, and its effective management is essential to reduce overall morbidity and mortality (147). The Study of Heart and Renal Protection (SHARP), which enrolled 9,270 individuals with moderate-to-advanced CKD, demonstrated that combination lipid-lowering therapy with simvastatin and ezetimibe reduced the incidence of major atherosclerotic events by 17% (148). A meta-analysis of 33 randomized controlled trials (RCTs) further confirmed the renoprotective effects of statins, showing reductions in urinary albumin excretion [weighted mean difference (WMD): −2.04 mg/24 h], proteinuria (WMD: −0.58 g/24 h), and modest improvements in creatinine clearance (WMD: +0.86 ml/min) (149). Based on these findings, current clinical guidelines recommend maintaining low-density lipoprotein cholesterol (LDL-C) levels below 100 mg/dl, with lower targets for individuals at elevated cardiovascular risk (150). Lipid-lowering therapy, particularly with statins and combination regimens, thus plays a central role in the comprehensive management of DKD.

### 3.4 Lifestyle modifications in DKD therapy

Lifestyle modification represents a critical adjunct to pharmacologic therapy in DKD management, offering substantial benefits for blood pressure control, metabolic regulation, and renal outcomes (151). Among dietary interventions, sodium restriction has the strongest evidence base. A Cochrane systematic review reported mean reductions of 6.15 mmHg in systolic and 3.41 mmHg in diastolic blood pressure following dietary sodium restriction (152). Current international guidelines recommend limiting sodium intake to 2.0–2.3 g/day (153). Observational data consistently demonstrate a strong correlation between sodium intake and albuminuria severity (154). Weight management also exerts important renoprotective effects. Mendelian randomization studies have identified a causal relationship between increased body mass index (BMI) and heightened kidney disease risk, with each standard deviation increase in BMI nearly doubling disease probability (odds ratio: 2.00) (155). Bariatric and metabolic surgeries have been associated with marked improvements in renal function and reductions in albuminuria among obese patients with DKD (156). Physical activity offers additional benefit. Structured exercise programs, particularly those incorporating resistance training, have been shown to improve glomerular filtration rates and reduce proteinuria (157). Current clinical guidelines recommend that DKD patients engage in at least 150 min per week of moderate-intensity aerobic exercise to support cardiorenal health (158).

### 3.5 Modern pharmacological approach

The contemporary pharmacologic framework for DKD emphasizes the use of four core medication classes, each contributing distinct yet complementary cardiorenal protective effects. These include: (1) RAASi—such as ACEIs and ARBs; (2) SGLT2i; (3) GLP-1 RAs; and (4) ns-MRAs (159, 160). Each class targets different pathogenic mechanisms—hemodynamic dysregulation, hyperglycemia, inflammation, and fibrosis—and has independently demonstrated benefits for renal and cardiovascular outcomes in randomized controlled trials. When used in combination, these agents exhibit additive and, in some cases, synergistic effects, offering a robust strategy for mitigating DKD progression and associated cardiovascular morbidity (161). The integration of these therapies into routine clinical practice represents a paradigm shift from glucose-centric models toward a comprehensive cardiorenal-metabolic treatment approach.

### 3.6 Integrated team-based care approach

Optimal management of DKD requires a multidisciplinary, team-based approach that leverages the expertise of various healthcare professionals. Primary care providers play a foundational role in screening, risk stratification, early intervention, and referral of high-risk individuals (162). Endocrinologists contribute specialized expertise in glycemic management, particularly in selecting agents with proven cardiorenal benefits, such as SGLT2i and GLP-1 RAs (163). As DKD advances, nephrologists take on a central role in managing progressive renal function decline, persistent albuminuria, complex electrolyte disturbances, and planning for renal replacement therapy when appropriate (164). Given the elevated cardiovascular burden in DKD, cardiologists are increasingly integral to the care team (165). However, data suggest that SGLT2 inhibitors and GLP-1 receptor agonists remain underutilized in cardiology practice despite their established benefits (166). Reframing these agents as foundational cardiorenal protective therapies, rather than merely glucose-lowering drugs, is essential to enhance their uptake across disciplines. Implementation of patient-centered care models—featuring integrated electronic health records, effective inter-provider communication, and collaborative clinical decision-making—remains critical to achieving therapeutic goals. Both the Kidney Disease: Improving Global Outcomes (KDIGO) guidelines and the American Diabetes Association (ADA) endorse multidisciplinary care coordination as a central pillar of comprehensive DKD management (167).

## 4 Medication-based therapeutic approaches for DKD

### 4.1 Inhibitors of the renin–angiotensin–aldosterone system (RAASi)

Blockade of the RAAS remains the foundational therapy for DKD, supported by robust evidence from landmark randomized controlled trials. The Collaborative Study Group (CSG) trial enrolled 409 patients with type 1 diabetes and persistent proteinuria (≥500 mg/day), randomizing them to captopril or placebo (168). Captopril reduced the risk of serum creatinine doubling by 48% (*P* = 0.007), with a more pronounced 76% risk reduction observed in patients with baseline serum creatinine >2.0 mg/dl. Moreover, captopril therapy halved the combined risk of mortality, dialysis initiation, or kidney transplantation. In patients with type 2 diabetes, the RENAAL trial evaluated 1,513 participants with overt nephropathy (169). Losartan treatment resulted in a 16% relative reduction in the composite outcome of doubling of serum creatinine, end-stage renal disease (ESRD), or death (*P* = 0.02). Notably, losartan decreased the risk of serum creatinine doubling and ESRD by 25 and 28%, respectively, and reduced proteinuria by 35%, compared to a 4% increase in the placebo group. The Irbesartan Diabetic Nephropathy Trial (IDNT) compared irbesartan to amlodipine and placebo in 1,715 patients with type 2 DKD (170). Irbesartan reduced the primary composite endpoint by 23% vs. amlodipine and 20% vs. placebo (both *P* < 0.01), while also slowing the rate of renal function decline (171, 172). Despite the strength of these findings, several limitations must be acknowledged. Differences in blood pressure between treatment arms may confound interpretation of direct renoprotective effects. Sample sizes limited secondary endpoint analysis, and pharmaceutical sponsorship introduces the possibility of selective outcome reporting. Additionally, composite endpoints combining irreversible outcomes with surrogate markers complicate interpretation. Nevertheless, these trials provide compelling and consistent evidence that RAAS inhibition reduces the progression of DKD and underpins current clinical guideline recommendations.

### 4.2 Inhibitors of sodium–glucose cotransporter 2 (SGLT2i)

SGLT2i represent a transformative advance in the management of CKD, including DKD, with efficacy demonstrated across multiple large-scale randomized trials. The CREDENCE trial enrolled 2,627 patients with type 2 diabetes and albuminuric kidney disease [urinary albumin-to-creatinine ratio (UACR) 300–5,000 mg/g; estimated glomerular filtration rate (eGFR) 30–90 ml/min/1.73 m^2^], randomizing participants to canagliflozin (100 mg daily) or placebo (173). Over a median 2.6-year follow-up, canagliflozin reduced the risk of the primary composite outcome—ESRD, doubling of serum creatinine, or death from renal or cardiovascular causes—by 30% (HR 0.70; 95% CI 0.59–0.82). The DAPA-CKD trial extended these findings to both diabetic and non-diabetic CKD populations. Among 4,304 participants (67.5% with diabetes), dapagliflozin (10 mg daily) reduced the primary composite outcome by 39% (HR 0.61; 95% CI 0.51–0.72) (174). Similarly, the EMPA-KIDNEY trial, which included 6,609 participants across various CKD etiologies and stages, demonstrated that empagliflozin reduced the risk of kidney disease progression or renal death by 28% (HR 0.72; 95% CI 0.64–0.82) (175). Despite their robust benefits, several caveats apply. All three trials were industry-sponsored, raising the potential for reporting bias. Importantly, patients with advanced renal impairment (eGFR < 20–30 ml/min/1.73 m^2^) were excluded, limiting generalizability to this high-risk subgroup. Moreover, long-term safety data in patients with severely reduced kidney function remain incomplete. Nonetheless, these studies firmly establish SGLT2 inhibitors as cornerstone therapy for DKD and broader CKD management. Their renoprotective effects—largely independent of glycemic control—have redefined treatment paradigms, expanding their role from antidiabetic agents to central components of cardiorenal protection across diverse patient populations.

### 4.3 Antagonists of mineralocorticoid receptors (MRAs)

MRAs have emerged as a novel and promising class of therapeutic agents for DKD. Finerenone, a selective non-steroidal MRA, has led this advancement by targeting aldosterone-mediated inflammatory and fibrotic signaling pathways—key drivers of DKD progression. Its mechanism of action complements those of SGLT2 inhibitors and RAAS blockers, contributing to a multifaceted therapeutic approach (176). The FIDELIO-DKD trial provided pivotal evidence supporting the efficacy of finerenone in patients with type 2 diabetes and CKD (177). In this randomized, placebo-controlled study of 5,734 participants (UACR 30–5,000 mg/g; eGFR 25–75 ml/min/1.73 m^2^), finerenone (10–20 mg daily) significantly reduced the primary composite renal outcome—defined as kidney failure, sustained eGFR decline ≥40%, or renal death—by 18% compared to placebo (HR 0.82; 95% CI 0.73–0.93; *P* = 0.001) over a median follow-up of 2.6 years. The FIGARO-DKD trial, which enrolled patients with earlier-stage DKD and preserved eGFR, demonstrated a 13% relative reduction in major adverse cardiovascular events (MACE; HR 0.87; 95% CI 0.76–0.98; *P* = 0.03), primarily driven by a 29% reduction in heart failure hospitalizations (178). The FIDELITY pooled analysis (*n* = 13,026) integrated data from both trials, confirming consistent cardiorenal benefits: a 23% reduction in the composite kidney outcome (HR 0.77; 95% CI 0.67–0.88) and an 18% reduction in all-cause mortality (HR 0.82; 95% CI 0.70–0.95) (179). Despite these encouraging findings, several limitations warrant consideration. All three trials were sponsored by the manufacturer (Bayer), raising the possibility of reporting bias. Hyperkalemia was a notable adverse event, occurring in 21.4% of finerenone-treated patients vs. 9.1% in the placebo group, necessitating close monitoring of serum potassium levels. Furthermore, the exclusion of patients with symptomatic heart failure may restrict the generalizability of results to this particularly vulnerable subgroup. Nevertheless, finerenone has firmly established itself as a critical component of contemporary DKD therapy. When combined with SGLT2 inhibitors, it enables a complementary, mechanism-based approach that targets multiple pathogenic pathways. These developments underscore a paradigm shift toward multi-targeted precision therapy in DKD, aligning with the broader goals of individualized medicine in nephrology.

### 4.4 Incretin-based therapies

GLP-1 RAs have emerged as promising agents in the management of DKD, exerting multifaceted benefits through improved glycemic control, weight reduction, anti-inflammatory effects, and modulation of renal hemodynamics (1). An expanding body of evidence from both cardiovascular outcome trials and kidney-specific studies supports their implementation in DKD care. In the LEADER trial, which enrolled 9,340 individuals with type 2 diabetes and elevated cardiovascular risk, liraglutide treatment resulted in a 22% reduction in a secondary composite renal outcome (HR 0.78; 95% CI 0.67–0.92), primarily due to reduced incidence of new-onset macroalbuminuria (180–182). Similarly, the SUSTAIN-6 trial (*n* = 3,297) reported a 36% relative reduction in kidney disease progression with semaglutide (HR 0.64; 95% CI 0.46–0.88) (183). Although both trials evaluated renal outcomes as secondary endpoints, the results provided compelling early evidence of renoprotection. The FLOW trial was the first randomized controlled trial specifically designed to evaluate renal outcomes with GLP-1 RA therapy (184). Among 3,534 participants with DKD (eGFR 25–75 ml/min/1.73 m^2^; UACR 100–5,000 mg/g), once-weekly semaglutide (1.0 mg) reduced the risk of the primary composite renal endpoint—defined as kidney failure, sustained ≥50% eGFR decline, or death from renal or cardiovascular causes—by 24% (HR 0.76; 95% CI 0.66–0.88) (185). Additionally, semaglutide reduced all-cause mortality by 20% (HR 0.80; 95% CI 0.67–0.95). The trial was terminated early due to clear efficacy, marking a major advancement in renal protection through incretin-based therapy. However, subgroup analyses from FLOW raised important questions regarding combination therapy. Among 550 participants concurrently receiving SGLT2 inhibitors, semaglutide conferred no significant renal benefit (HR 1.07; 95% CI 0.69–1.67), in contrast to substantial protection observed in those not on SGLT2i therapy (HR 0.73; 95% CI 0.63–0.85). These findings suggest a potential therapeutic ceiling when combining agents with overlapping mechanisms—including natriuresis, afferent arteriolar constriction, and anti-inflammatory effects—and underscore the need for further investigation into optimal sequencing and drug combinations. Several caveats must be considered when interpreting these data. All major GLP-1 RA trials—including LEADER, SUSTAIN-6, and FLOW—were sponsored by pharmaceutical companies (Novo Nordisk or Eli Lilly), raising the possibility of reporting bias. Except for FLOW, renal outcomes were evaluated as secondary endpoints, with limited statistical power for definitive conclusions. Moreover, patients with advanced kidney dysfunction (eGFR < 25 ml/min/1.73 m^2^) were consistently excluded, limiting applicability to this high-risk population. Long-term safety and efficacy in patients with severe renal impairment remain to be fully characterized. In summary, GLP-1 receptor agonists now represent a key therapeutic option in the comprehensive management of DKD. Their distinct mechanisms of action complement those of RAASi and SGLT2i, offering additional opportunities for personalized care. Future research should focus on identifying optimal therapeutic combinations and addressing unmet needs in underrepresented and high-risk patient groups.

### 4.5 Inhibitors of dipeptidyl peptidase-4 (DPP-4)

Dipeptidyl peptidase-4 (DPP-4) inhibitors exert their effects by blocking DPP-4 enzymatic activity, thereby preventing the degradation of endogenous incretin hormones—primarily glucagon-like peptide-1 (GLP-1) and glucose-dependent insulinotropic peptide (GIP). This prolongs the bioactivity of incretins, contributing to glucose-dependent insulin secretion and suppression of glucagon release. Beyond their glycemic effects, DPP-4 inhdiabetic kidney diseaseibitors have shown potential renoprotective properties, including improved renal perfusion, attenuation of oxidative stress, reduced proinflammatory signaling, and enhanced tubular epithelial cell viability (186). In a clinical study by Kim and colleagues involving 414 patients with DKD, treatment with DPP-4 inhibitors led to a 28.4% reduction in UACR, compared with only an 8.2% reduction in the control group (*P* < 0.01) (186). The annual rate of decline in estimated glomerular filtration rate (eGFR) was also slower among treated individuals (1.24 vs. 2.01 ml/min/1.73 m^2^; *P* = 0.03), indicating modest renoprotective benefit. DPP-4 inhibitors offer distinct clinical advantages, particularly in high-risk populations. They are weight-neutral, carry a low risk of hypoglycemia, and are well-tolerated, with rare gastrointestinal side effects (187). These features make them suitable for elderly patients, those with impaired renal function, and individuals intolerant to other glucose-lowering agents. However, their cardiorenal efficacy is modest compared with that of SGLT2i and GLP-1 receptor agonists. As a result, current treatment guidelines position DPP-4 inhibitors as secondary options—primarily for patients who cannot tolerate or are ineligible for first-line renoprotective therapies (1). Although they do not offer the same magnitude of benefit as newer agents, DPP-4 inhibitors remain valuable in individualized DKD management. Further studies are warranted to evaluate their role in combination regimens alongside other evidence-based therapies. An overview of these pivotal trials, including inclusion criteria and primary/secondary endpoints, can be found in Table 1.

**Table 1:** This Table provides a comprehensive overview of landmark randomized controlled trials (RCTs) in DKD, structured according to the PICO format: Population (P), Intervention (I), Comparison (C), Outcome (O).

**Trial name**	**Population**	**Intervention**	**Comparison**	**Primary outcomes**
Captopril Trial (1993)	Type 1 diabetes with nephropathy	Captopril (ACEI)	Placebo + other antihypertensives	SlowerCrCldecline; 50%↓ ESRD/transplant/death
RENAAL (2001)	T2DM + proteinuria	Losartan (ARB)	Place·bo + standard care	16% ↓ doubling SCr; 28% ↓ ESRD
IDNT (2001)	T2DM + HTN + nephropathy	Irbesartan (ARB)	Amlodipine or placebo	20% ↓ doubling SCr; 23% ↓ ESRD
CREDENCE (2019)	T2DM + eGFR 30– < 90 +albuminuria >300mg/g	Canagliflozin + RAASi	Placebo + RAASi	30% ↓ renal/Coutcome; trial stopped early
DAPA-CKD (2020)	CKD (eGFR 25–75) ± diabetes	Dapagliflozin+SOC	Placebo + SOC	44% ↓ in renal/CV composite outcome
EMPA+KIDNEY (2023)	CKD with/without diabetes, eGFR ≥20	Empagliflozin+SOC	Placebo + SOC	28% ↓ ESKD/CV death; 14% ↓ hospitalization
FIDELIO-DKD (2020)	T2DM + CKD	Finerenone + RASi	Placebo + RASi	18% ↓ renal outcome; 14% ↓ CV events
FIGARO-DKD (2021)	T2DM + early CKD	Finerenone + RASi	Placebo + RASi	13% ↓ CV outcome, esp. HF hospitalization
FIDELITY (2022)	Pooled FIDELIO + FIGARO	Finerenone + RASi	Placebo + RASi	23% ↓ renal, 14% ↓ CV composite
LEADER (2016)	T2DM + high CV risk	Liraglutide	Placebo	22% ↓ CV death; ↓ nephropathy
SUSTAIN-6 (2016)	T2DM + high CV risk	Semaglutide	Placebo	36% ↓ nephropathy; ↓ eGFR decline
AMPLITUDE-O (2021)	T2DM + CKD or CVD	Efpeglenatide	Placebo	32% ↓ composite renal outcome
FLOW (ongoing)	T2DM + CKD	Semaglutide	Placebo	Renal endpoints (pending)

Trials are categorized by drug class, and outcomes are labeled as Primary or Secondary endpoints. Key inclusion criteria are summarized for clarity.

T1DM, type 1 diabetes mellitus; T2DM, type 2 diabetes mellitus; CKD, chronic kidney disease; eGFR, estimated glomerular filtration rate; UACR, urinary albumin-to-creatinine ratio; ESRD, end-stage renal disease; CV, cardiovascular; ACEI, angiotensin-converting enzyme inhibitor; ARB, angiotensin II receptor blocker; SGLT2i, sodium-glucose cotransporter-2 inhibitor; GLP-1RA, glucagon-like peptide-1 receptor agonist; GIP, glucose-dependent insulinotropic polypeptide; MRA, mineralocorticoid receptor antagonist; Non-steroidal MRA, selective non-steroidal mineralocorticoid receptor blocker; ERA, endothelin receptor antagonist; HF, heart failure.

### 4.6 Phosphodiesterase inhibitors

Pentoxifylline, a non-selective phosphodiesterase inhibitor, has shown modest clinical benefit in patients with moderate to advanced stages (3, 4) of DKD. Its therapeutic effects are attributed to multiple mechanisms, including upregulation of the renoprotective Klotho protein, suppression of proinflammatory cytokines, attenuation of tissue fibrosis, and reduction of proteinuria (188). These actions collectively contribute to the preservation of renal function and deceleration of disease progression. Pentoxifylline is generally well-tolerated. The most frequently reported adverse effects are gastrointestinal in nature—nausea, vomiting, and abdominal discomfort (189). Although bleeding complications are rare, caution is warranted in patients with recent cerebral or retinal hemorrhage due to its potential antiplatelet activity (190). Given its low cost, pleiotropic effects, and favorable safety profile, pentoxifylline may serve as a valuable adjunctive treatment in select patients with progressive DKD. It offers a cost-effective therapeutic addition, particularly in resource-limited settings or among patients unable to access newer agents. However, broader clinical adoption will require further validation through large-scale, randomized trials.

### 4.7 Therapeutic approaches targeting advanced glycation end products (AGEs)

AGEs comprise harmful compounds formed when proteins and lipids undergo non-enzymatic glycation in hyperglycemic environments. These molecules promote tissue damage by inducing oxidative stress and chronic inflammatory responses, significantly contributing to DKD progression and other diabetic complications, including cardiovascular disorders (191). Emerging research suggests that dietary modifications aimed at limiting AGEs consumption can improve insulin sensitivity, reduce glycemic parameters, and decrease systemic inflammatory markers (192). While pharmaceutical agents that inhibit AGEs formation remain in early developmental stages, nutrition-based interventions to minimize AGEs exposure represent practical, economical, and beneficial therapeutic approaches that complement comprehensive DKD management (193). Future investigations should focus on defining recommended dietary AGEs intake limits and thoroughly assessing their long-term clinical effects, facilitating the incorporation of targeted nutritional strategies into routine clinical recommendations.

### 4.8 Vitamin D-related compounds

Active vitamin D (calcitriol) and related synthetic analogs function as steroid-like hormones that regulate mineral homeostasis, immune function, and cellular differentiation. CKD patients frequently exhibit vitamin D insufficiency, which strongly correlates with accelerated renal function decline and increased albuminuria (194, 195). Administration of active vitamin D consistently improves endothelial function, reduces albuminuria, and inhibits progression of renal fibrosis (196–198). Mechanistic studies demonstrate that vitamin D receptor (VDR) activation reduces podocyte apoptosis by suppressing NF-κB signaling cascades (199, 200). Paricalcitol, a selective vitamin D receptor activator, has shown promising results in preclinical research by modulating Wnt signaling pathways, reducing glucose-induced podocyte injury, and markedly decreasing albumin excretion (201). In a controlled clinical trial involving 45 patients with persistent albuminuria despite optimal renin-angiotensin-aldosterone system blockade, paricalcitol treatment achieved significant additional reductions in albuminuria compared with placebo (202). These observations support VDR targeting as a promising adjunctive therapeutic strategy for DKD, particularly among high-risk individuals with residual albuminuria, offering an innovative approach beyond conventional management.

## 5 Integrated therapeutic approaches

The pathophysiology of DKD is multifactorial, involving hyperglycemia, intraglomerular hypertension, chronic inflammation, oxidative stress, and progressive fibrosis. These interconnected pathways necessitate treatment strategies that extend beyond monotherapy. Contemporary DKD management increasingly emphasizes the use of complementary pharmacologic agents targeting distinct mechanisms to achieve broader and more durable therapeutic effects. A growing body of evidence supports the use of multi-agent regimens combining RAASi, SGLT2i, GLP-1 RAs, and ns-MRAs. These combinations have demonstrated superior efficacy in reducing albuminuria, preserving glomerular filtration, and mitigating cardiovascular events compared with individual drug classes alone. As mechanistic insights and pharmacologic innovation continue to expand, the DKD treatment paradigm is shifting toward personalized, multi-targeted therapeutic strategies. Tailored regimens, optimized through emerging biomarker data and patient-specific risk profiling, hold promise for enhancing both renal and cardiovascular outcomes in future clinical practice.

### 5.1 Comprehensive strategy combining multiple medication classes

A recent actuarial modeling study assessed the potential clinical benefits of combining three major pharmacologic classes—SGLT2 inhibitors, GLP-1 receptor agonists, and the non-steroidal MRA finerenone—using data from large-scale randomized trials (161). The analysis integrated findings from the CANVAS and CREDENCE trials (*n* = 14,543) for SGLT2i (203), a meta-analysis of eight cardiovascular outcome trials for GLP-1 RAs (*n* = 60,080) (204), and the FIDELIO-DKD and FIGARO-DKD studies for finerenone (*n* = 13,026) (179). Results were striking. The three-drug combination was projected to reduce major adverse cardiovascular events (MACE) by 35% (HR 0.65; 95% CI 0.55–0.76), decrease hospitalization for heart failure by 68%, and slow CKD progression by 58% (HR 0.42; 95% CI 0.31–0.56). In hypothetical patients aged 50 years, this regimen could extend MACE-free survival by 3.2 years and delay kidney disease progression by 5.5 years. Despite the compelling projections, several important limitations must be acknowledged. No clinical trial to date has directly evaluated triple therapy vs. single or dual combinations; these findings are derived from indirect comparisons that assume independent and additive effects across agents. All source studies were industry-sponsored, introducing potential bias due to selective reporting. Additionally, the simulation relied on clinical trial populations that may not fully reflect real-world patient heterogeneity, possibly overestimating effectiveness. Furthermore, mechanistic overlap—particularly in anti-inflammatory and natriuretic effects—raises the possibility of diminishing returns when combining these agents. Nevertheless, this analysis offers robust support for advancing comprehensive, multi-target treatment strategies in DKD. As therapeutic options expand and evidence accumulates, structured combination therapy—especially when guided by biomarker-driven risk stratification—has the potential to redefine DKD management and optimize long-term cardiorenal protection.

### 5.2 Renal triple therapy for kidney protection

The therapeutic strategy combining RAASi, SGLT2i, and ns-MRAs—collectively referred to as Renal Triple Therapy (RTT)—offers synergistic, mechanism-based protection in the management of DKD. This multi-drug approach concurrently targets intraglomerular hypertension, tubular inflammation, and fibrotic remodeling (1, 177, 178, 205). A recent network meta-analysis reported that RTT reduced the risk of cardio-renal composite outcomes by 46% compared to RAASi monotherapy (HR 0.54; 95% CI 0.50–0.58) (206). The FIDELITY analysis, which pooled data from the FIDELIO-DKD and FIGARO-DKD trials (*n* = 13,026; median follow-up: 3.0 years), demonstrated that finerenone reduced cardiovascular events by 14% (HR 0.86; *P* = 0.0018), kidney events by 23% (HR 0.77; *P* = 0.0002), and all-cause mortality by 18% (HR 0.82; *P* = 0.014) (179, 207). Importantly, patients receiving concurrent SGLT2i therapy experienced even greater renoprotection, with a 58% reduction in kidney composite outcome risk (HR 0.42) (208). Despite these compelling findings, several barriers hinder widespread clinical adoption of RTT. Hyperkalemia remains a primary safety concern. In FIDELIO-DKD, mild hyperkalemia (serum potassium ≥5.5 mmol/L) occurred in 21.4% of finerenone-treated patients compared to 9.2% in the placebo group (209). While SGLT2 inhibitors may help mitigate this risk, implementation of rigorous potassium monitoring protocols remains essential when combining agents (210). Real-world applicability is another limitation. Pivotal trials systematically excluded certain patient populations: 43.2% of individuals with CKD without albuminuria and 6.9% with eGFR below 25 ml/min/1.73 m^2^—two cohorts frequently encountered in clinical practice (211). Future research should focus on determining the optimal sequencing of these agents, characterizing long-term safety and tolerability in diverse populations, and developing personalized, biomarker-driven treatment algorithms. Such efforts will be critical to fully realize the therapeutic potential of Renal Triple Therapy and integrate it effectively into standard care pathways for DKD.

### 5.3 Combining eastern and western medical approaches for DKD treatment

#### 5.3.1 Single-agent therapies from traditional Chinese medicine

Within the therapeutic framework for DKD, single-agent remedies derived from TCM can be broadly classified into three functional categories based on their pharmacological actions: tonic preparations, antipyretic substances, and agents that activate blood circulation and resolve stasis. Tonic preparations are primarily used to reinforce kidney function and correct metabolic disturbances. *Cordyceps sinensis* (caterpillar fungus) and its active constituents have shown multiple beneficial effects in DKD models, including glucose-lowering, anti-inflammatory, antioxidant, antifibrotic, immunomodulatory, and cytoprotective activities (212). These properties have been incorporated into various patented TCM formulations that demonstrate measurable clinical efficacy. Recent studies have highlighted astragaloside IV (AS-IV)—the principal bioactive component of *Astragalus membranaceus*—as a particularly promising candidate. AS-IV mitigates DKD-associated renal injury by inhibiting ferroptosis in tubular epithelial cells, primarily through suppression of the HIF-1α/HMOX1 signaling pathway. It also enhances glucose and lipid metabolism by upregulating protective proteins such as glutathione peroxidase 4 (GPX4) and ferritin heavy chain 1, while downregulating ferroptosis-related mediators including ACSL4 and transferrin receptor 1 (213). In related research, a compound mixture (MIX) comprising three bioactive flavonoids—baicalin, wogonin, and wogonoside—derived from *Scutellaria baicalensis* has been shown to attenuate renal fibrosis in diabetic models by inhibiting activation of the TGF-β/Smads signaling cascade. Additionally, this formulation improved metabolic indices and ameliorated structural kidney damage. Collectively, these results provide mechanistic validation and preclinical support for the development of innovative DKD therapies based on well-characterized herbal constituents (214). These findings underscore AS-IV's potential as an innovative anti-ferroptotic therapeutic agent. Antipyretic substances exert renoprotective effects primarily via scavenging of ROS and suppression of inflammatory responses. *Pueraria lobata*-derived flavonoids, including puerarin, enhance antioxidant enzyme activities—such as superoxide dismutase (SOD) and catalase (CAT)—reduce accumulation of AGEs, and attenuate oxidative damage and apoptosis (215). Similarly, gypenosides, bioactive saponins from *Gynostemma pentaphyllum*, exhibit potent lipid-lowering activity and modulate gut microbiota composition in high-fat diet-fed rats, suggesting potential gut–kidney axis-mediated metabolic benefits in DKD (216). Circulation-activating, stasis-resolving agents have shown promise in attenuating renal fibrosis and regulating immune responses. *Tripterygium wilfordii* has demonstrated significant efficacy in preclinical DKD models. Network pharmacology and molecular docking analyses identified 29 active compounds that target 134 potential proteins—63 of which are directly implicated in DKD pathophysiology. Protein–protein interaction network analysis highlights TNF and AKT1 as central regulatory nodes, suggesting that Tripterygium may modulate key inflammatory and fibrotic signaling pathways (217). *Panax notoginseng* is widely used as an adjunct therapy in DKD. A comprehensive meta-analysis encompassing 1,918 participants from 24 randomized controlled trials found that combining *Panax notoginseng* with standard care significantly reduced urinary albumin excretion (mean difference −26.89 mg), 24-h proteinuria (−0.32 g), and serum creatinine (−4.52 μmol/L) (218). Improvements were also observed in lipid parameters: total cholesterol (TC) (−1.56 mmol/L), triglycerides (−0.56 mmol/L), and low-density lipoprotein cholesterol (−0.94 mmol/L), supporting its utility as a complementary metabolic regulator in DKD management. Additionally, osthole—a natural coumarin compound derived from *Cnidium monnieri*—has shown promise in early-stage DKD by downregulating components of the TGF-β1/Smads signaling cascade, thereby attenuating the progression of glomerulosclerosis (219). Ultra-high-performance liquid chromatography coupled with quadrupole time-of-flight mass spectrometry (UHPLC-QTOF-MS) identified 26 chemical constituents in serum following administration of Alpiniae oxyphyllae fructus (AOF). Integrating network pharmacology with molecular docking analysis, five candidate bioactive compounds—including Cubebin and dihydrochalcone derivatives—were identified as targeting key pathogenic proteins such as TP53, SRC, STAT3, PIK3CA, and AKT1 (220). This study systematically elucidated the mechanism by which AOF modulates cellular senescence in DKD, progressing from compound identification and core target prediction to functional validation in both *in vitro* and *in vivo* models. These findings provide both a mechanistic rationale and experimental evidence supporting the potential incorporation of AOF into modern therapeutic frameworks. Researchers have developed and validated AANG—a botanical formulation comprising asiatic acid (a Smad7 activator) and naringenin (a Smad3 inhibitor) that attenuates renal fibrosis and inflammation in DKD through modulation of the TGF-β/Smad3/Smad7 signaling pathway. In db/db mouse models, AANG treatment preserved renal architecture, improved kidney function, and promoted pancreatic β-cell proliferation, thereby enhancing insulin secretion and restoring glucose and lipid homeostasis (221). Notably, AANG delayed the onset of type 2 diabetes (T2D) in prediabetic mice, indicating its dual potential for both prevention and treatment of T2D and its renal complications. These findings offer compelling therapeutic insights into multi-target interventions rooted in Traditional Chinese Medicine (TCM) principles for managing diabetes and its associated nephropathy (222). In summary, several TCM-derived single-agent therapies exhibit notable potential as adjunctive interventions in DKD management. Future studies should focus on elucidating precise mechanisms of action, optimizing dosing strategies, and validating efficacy and safety through rigorously designed randomized controlled trials (RCTs).

#### 5.3.2 Traditional Chinese medicine for addressing intestinal microbial imbalance in DKD

TCM has shown notable potential in regulating gut–kidney axis dysfunction, offering a novel therapeutic avenue for managing DKD. A growing body of experimental evidence links DKD pathogenesis and progression with intestinal dysbiosis, gut barrier disruption, and elevated levels of gut-derived microbial toxins (223). Multiple TCM formulations have demonstrated efficacy in restoring intestinal microbial homeostasis while attenuating renal injury. QiDiTangShen Granules (QDTS), for example, have been shown to restructure intestinal bacterial communities and modulate bile acid metabolism in db/db mice (224). These changes activate the microbiota–bile acid–farnesoid X receptor (FXR) signaling pathway, leading to significant reductions in renal damage. Likewise, Yishen Huashi Granules (YSHS) preserve renal function via modulation of the microbiota–metabolism–transcriptome network (225). In streptozotocin-induced DKD rat models, YSHS increased the abundance of beneficial Lactobacillus species and reduced potentially pathogenic Prevotella populations (226). Other studies have further substantiated the role of TCM in counteracting intestinal dysbiosis. Anemarrhena asphodeloides extract (AAE) improves microbial diversity and promotes the growth of beneficial bacteria such as Blautia coccoides (227). By enhancing peroxiredoxin activity, AAE supports gut–pancreas crosstalk and contributes to improved glycemic regulation (228). Additional formulations, including Qingre Xiaozheng Formula and Tang-Shen-Fang, have demonstrated the ability to rebalance microbial ecosystems, suppress metabolic inflammation, and reduce the production of gut-derived uremic toxins, thereby conferring renoprotective effects (229, 230). In another study, a formulation containing four Bifidobacterium strains significantly reduced total cholesterol (TC) levels and downregulated interleukin-2 (IL-2) expression—a key pro-inflammatory cytokine—in patients with DKD, indicating potential lipid-lowering and anti-inflammatory benefits. In parallel, treatment with Jingui Shenqi Pill produced more pronounced improvements in TCM syndrome scores and greater reductions in the urine albumin-to-creatinine ratio (ACR). Microbiota analysis revealed that the probiotic intervention selectively increased the abundance of Prevotella_7, potentially enhancing carbohydrate metabolism and systemic energy balance. Although neither intervention significantly altered global microbial diversity indices, the targeted modulation of specific taxa supports the feasibility of precision microbiome-based therapies for DKD. Collectively, these findings strengthen the rationale for developing multifaceted strategies that leverage intestinal microbiota modulation to mitigate DKD progression (231).

Recent studies have demonstrated that *Abelmoschus manihot* extract, marketed as Huangkui Capsule (HKC), attenuates diabetic nephropathy progression by reshaping gut microbiota composition, optimizing metabolic parameters, and suppressing expression of renal pro-inflammatory genes. These effects appear to be mediated through the gut–kidney axis, reinforcing the hypothesis that Traditional Chinese Medicine (TCM) agents exert systemic, organ-crosstalk effects. This mechanistic insight provides a modern conceptual basis for integrating TCM approaches into DKD management (232). TCM-based interventions targeting the intestinal microbiota represent mechanistically plausible and clinically promising strategies for DKD treatment. Future research should explore integrative approaches that combine microbial modulation with immune and metabolic regulation, potentially enabling personalized and multi-pathway therapeutic strategies for patients with DKD.

#### 5.3.3 Evidence from multicenter trials supporting combined TCM and RAASi therapy

Recent years have seen the emergence of multicenter randomized controlled trials (RCTs) evaluating the efficacy of combining TCM formulations with RAASi in the treatment of DKD. One multicenter trial involving 413 participants found that combining irbesartan with Huangkui Capsules led to a significant reduction in urinary albumin-to-creatinine ratio (UACR), with a mean decrease of 262.3 mg/g, compared to 89.1 mg/g with irbesartan alone (*P* < 0.001) (233). Similarly, a clinical trial assessing Keluoxin Capsules (*n* = 129) demonstrated that co-administration with standard therapy significantly reduced log-transformed UACR vs. placebo (*P* = 0.029) and decreased the risk of ≥30% UACR elevation by 74% (234). Despite these encouraging findings, several limitations must be acknowledged. Most trials had short treatment durations, typically limited to 24 weeks, precluding assessment of long-term outcomes such as progression to ESKD. Small sample sizes reduced statistical power, and methodological concerns—including insufficient reporting of randomization procedures (e.g., in the Huangkui Capsule trial)—limit the strength of conclusions. Furthermore, most studies relied on surrogate endpoints, such as albuminuria, without directly assessing renal function decline or definitive clinical events. Taken together, current evidence suggests that adding TCM preparations to RAAS-based therapy may enhance albuminuria reduction in patients with DKD. Another TCM-based formulation, Shenshuaining (SSN)—composed of extracts from *Astragalus membranaceus* (Huangqi), *Salvia miltiorrhiza* (Danshen), and *Rehmannia glutinosa* (Dihuang)—has demonstrated significant therapeutic efficacy in patients with DKD. Mechanistic investigations reveal that SSN improves renal function and reduces proteinuria by modulating immune responses, mitigating oxidative stress, and inhibiting renal fibrosis. Clinical data further indicate that combining SSN with either angiotensin-converting enzyme inhibitors (ACEIs) or angiotensin receptor blockers (ARBs) enhances renal protection compared to ACEI/ARB monotherapy. Such combination regimens significantly lower serum creatinine and blood urea nitrogen levels, suggesting a capacity to slow renal function decline (235). When added to standard care, SSN appears to augment renoprotective outcomes with an acceptable safety profile. Nonetheless, confirmation of its efficacy across heterogeneous patient populations will require large-scale, multicenter randomized controlled trials. The molecular structures of representative active compounds are presented in Table 2.

**Table 2:** The table illustrates the molecular structures of seven pharmacologically active phytochemicals identified in TCMs commonly applied in DKD management: Cordycepin (*Cordyceps sinensis*), Astragaloside IV (*Astragalus membranaceus*), Puerarin (*Pueraria lobata*), Gypenoside XVII (*Gynostemma pentaphyllum*), Triptolide (*Tripterygium wilfordii*), Notoginsenoside R1 (*Panax notoginseng*), and Osthole (*Cnidium monnieri*).

**Botanical name (Chinese name)**	**Extract type**	**Major active compound**	**Plant part used**	**Structural modifications**	**Pharmacological mechanisms**
*Cordyceps sinensis*	Water extract, fungal polysaccharides, nucleosides	Cordycepin	Fruiting body	Deoxyadenosine analog, non-glycosylated	Anti-inflammatory, antioxidant, anti-apoptotic, immunomodulatory
*Pueraria lobata*	Flavonoids	Puerarin	Root	C-glycosyl flavonoid	Antioxidant, anti-AGEs, SOD/CAT enhancement
*Gynostemma pentaphyllum*	Saponins (Gypenosides)	Gypenoside aglycones	Aerial parts	Steroidal backbone with glycoside chains	Hypolipidemic, gut microbiota regulation, NF-κB/TNF-α, insulin sensitivity
*Tripterygium wilfordii*	Ethanol extract, diterpenoids	Triptolide	Root bark	Epoxide and unsaturated rings	Anti-inflammatory, antifibrotic, targets TNF/AKT1 pathways
*Panax notoginseng*	Total saponins	Notoginsenoside R1	Rhizome	Triterpenoid backbone, glycoside conjugates	Reduce proteinuria, lipid regulation, RAAS synergy
*Cnidium monnieri*	Coumarins	Osthole	Fruit	Coumarin structure with isopentenyl side chain	Anti-oxidative, inhibits TGF-β1/Smads, anti-fibrotic
*Astragalus membranaceus*	Total saponins, ethanol extract	Astragaloside IV	Root	Multiple glycoside side chains	Multiple glycoside side chains

These compounds exert renoprotective effects through mechanisms such as inhibition of oxidative stress, inflammation, fibrosis, and ferroptosis, as well as modulation of metabolic and gut–renal pathways.

DKD, diabetic kidney disease; TCM, traditional Chinese medicine; AGEs, advanced glycation end products; SOD, superoxide dismutase; CAT, catalase; NF-κB/TNF-α, tumor necrosis factor-alpha/nuclear factor-kappaB; TNF/AKT1, tumor necrosis factor/threonine-proteinkinase; TGF-β1, transforming growth factor-beta 1; RAAS, renin–angiotensin–aldosterone system.

However, large-scale, high-quality RCTs with extended follow-up are essential to validate long-term renoprotective effects and assess impacts on clinically meaningful outcomes such as kidney failure, cardiovascular events, and mortality.

## 6 Future directions in DKD treatment

### 6.1 Targeting metabolic, inflammatory, and fibrotic pathways

The progression of DKD arises from intricate interactions between metabolic dysregulation, chronic inflammation, and renal fibrosis. Advances in pathophysiological understanding have revealed several promising molecular targets within these interrelated pathways, offering potential avenues for innovative therapeutic development. Endothelin receptor antagonists (ERAs) have shown renoprotective efficacy by inhibiting endothelin-mediated vasoconstriction and suppressing pro-inflammatory and pro-fibrotic signaling cascades (236). These effects translate into reductions in albuminuria, mitigation of fibrotic remodeling, and preservation of renal function (236–238). Similarly, inverse agonists of the cannabinoid-1 receptor have demonstrated favorable metabolic and structural outcomes in preclinical models, improving insulin sensitivity and maintaining renal architecture (239, 240). The transcription factor nuclear factor erythroid 2–related factor 2 (Nrf2) plays a central role in redox homeostasis and inflammation (241). Pharmacological activation of Nrf2 enhances the expression of cytoprotective genes, conferring resistance against oxidative injury and fibrogenesis in DKD models (242). Another emerging target is ceramide synthase 6 (CerS6), a key regulator of sphingolipid metabolism implicated in sterile inflammation. CerS6 promotes mitochondrial DNA release and activates the cGAS–STING pathway, amplifying inflammatory responses and aggravating kidney damage (243, 244). Inhibition of CerS6 has been shown to attenuate renal injury in experimental models, underscoring its therapeutic potential (244). Collectively, these mechanistic insights highlight the feasibility of multi-targeted therapeutic approaches that concurrently address metabolic abnormalities, inflammation, and fibrosis. Future efforts should focus on translating these findings into clinically viable interventions, with an emphasis on long-term efficacy, safety, and personalized application.

### 6.2 The intestinal microbiome as a therapeutic target

Emerging evidence supports the intestinal microbiome as a novel and modifiable target in the treatment of DKD. Accumulating data from clinical trials and meta-analyses suggest that probiotic supplementation may confer renoprotective benefits through modulation of gut microbial communities, metabolic regulation, and suppression of systemic inflammation. A meta-analysis of seven randomized controlled trials involving 456 patients demonstrated that probiotics significantly improved renal outcomes: eGFR increased [standardized mean difference (SMD) = 6.03; 95% CI: 0.84–11.21; *P* = 0.020], while serum creatinine and blood urea nitrogen levels declined (*P* < 0.05) (245). Oxidative stress markers also improved, with significant increases in glutathione and total antioxidant capacity (*P* < 0.001), and decreased malondialdehyde concentrations (*P* = 0.020) (245). Inflammatory markers such as high-sensitivity C-reactive protein were significantly reduced (*P* < 0.001) (245). Further support comes from a randomized, double-blind, controlled trial involving 60 DKD patients, in which daily administration of 8 × 10^9^ CFU probiotics for 12 weeks improved fasting glucose, HOMA-IR, and lipid profiles (*P* < 0.01) (246). A comprehensive 2023 Cochrane review evaluating 45 trials (*n* = 2,266) confirmed the safety of probiotic interventions, with most adverse events being mild gastrointestinal symptoms (247). According to GRADE methodology, evidence for improvements in blood urea nitrogen, total cholesterol, and LDL-C was of moderate quality, whereas data on serum creatinine, UACR, fasting glucose, and HbA1c were rated low quality (248). Despite promising findings, several limitations constrain the clinical applicability of existing data. Notably, there is substantial heterogeneity among trials in terms of DKD diagnostic criteria, probiotic strains, dosages, treatment durations, and outcome measures (98). Most studies enrolled small cohorts (30–200 participants) with short follow-up periods (8–24 weeks), precluding evaluation of hard clinical endpoints such as progression to ESKD (98, 247). Furthermore, the geographic concentration of study populations—primarily among Asian, particularly Chinese, patients—raises concerns regarding generalizability (98, 249). Mechanistic studies suggest that probiotics exert their effects through microbial rebalancing, augmentation of short-chain fatty acid-producing bacteria (e.g., Faecalibacterium, Roseburia), and reduction in uremic toxin production (98, 250). Moving forward, high-quality multicenter clinical trials with larger sample sizes, longer follow-up, and standardized endpoints are essential to validate efficacy. Personalized approaches based on host–microbiome interactions may further enhance therapeutic outcomes. Current evidence supports the adjunctive use of multi-strain probiotic formulations (≥4 × 10^9^ CFU daily for 8–12 weeks) in DKD management, provided they are administered under appropriate clinical supervision and integrated within a broader therapeutic framework.

### 6.3 Genetic approaches and cellular therapy

Stem cell–based interventions, particularly those utilizing mesenchymal stem cells (MSCs), hold considerable promise for the treatment of DKD, although clinical translation remains at an early stage. A meta-analysis of 40 preclinical studies involving 992 diabetic rodent models demonstrated that MSC-based therapies markedly improved renal function and attenuated key pathological features, including fibrosis, inflammation, apoptosis, and oxidative stress (251). Focused analysis of MSCs revealed potent glucose-lowering effects [standardized mean difference (SMD) = −1.954; 95% CI: −2.389 to −1.519; *P* < 0.001], as well as significant reductions in serum creatinine (SMD = −4.838; *P* < 0.001) and blood urea nitrogen (SMD = −4.912; *P* < 0.001), indicating substantial renoprotective benefits (252). The NEPHSTROM trial remains the most advanced human study to date. This phase 1b/2a randomized, placebo-controlled trial enrolled 16 patients with type 2 diabetes and progressive DKD, who were allocated in a 3:1 ratio to receive either a single intravenous infusion of 80 × 106 ORBCEL-M cells (CD362-selected, allogeneic bone marrow–derived MSCs) or placebo (253). The treatment exhibited a favorable safety profile, with no serious treatment-related adverse events reported. In terms of efficacy, the MSC-treated group exhibited a slower annual decline in eGFR. However, measured GFR did not differ significantly between groups, raising questions about the clinical relevance of this finding. Notably, MSC therapy preserved circulating regulatory T cells and stabilized inflammatory monocyte subsets, suggesting immunomodulatory effects. Despite encouraging preclinical and early clinical data, multiple translational hurdles remain. Considerable heterogeneity across animal studies (*I*^2^ = 85.1%−90.8%) arises from variability in cell sources, administration routes, dosing protocols, and outcome measures (252). The limited sample size in NEPHSTROM reduces statistical power and precludes definitive conclusions regarding efficacy (253). Furthermore, most animal models of DKD rely on chemically induced diabetes, which may inadequately reflect the multifactorial pathophysiology of human DKD (254). Mechanistic studies suggest that MSCs exert their benefits predominantly via paracrine signaling, including anti-inflammatory (e.g., downregulation of IL-6, IL-1β, and TNF-α), anti-fibrotic, and pro-angiogenic effects, as well as preservation of mitochondrial function (254–257). However, MSCs derived from patients with type 2 diabetes exhibit functional impairments, including accelerated senescence, impaired proliferation, and reduced therapeutic efficacy, thereby limiting the feasibility of autologous cell therapies (254). Critical barriers to clinical translation include the lack of standardized manufacturing protocols, variability in quality control, uncertainty regarding optimal delivery methods, and the absence of consensus on therapeutic dosing regimens. To advance MSC-based therapy toward routine clinical application in DKD, future research must prioritize large, well-powered, rigorously designed late-phase clinical trials, along with the development of predictive biomarkers for therapeutic responsiveness. Additionally, combination strategies that integrate MSCs with established pharmacological agents—such as SGLT2i—warrant exploration as potentially synergistic treatment modalities.

## 7 Summary and future directions

### 7.1 Clinical practice implications

This comprehensive review underscores several critical considerations for improving the clinical management of DKD. First, compelling evidence now supports a shift from traditional monotherapy toward multi-targeted combination regimens. Rather than incremental escalation, clinicians should consider early initiation of dual or triple therapy in patients with persistent albuminuria despite optimized RAAS inhibition. The FIDELITY pooled analysis demonstrated that Renal Triple Therapy (RTT) reduced the risk of composite kidney outcomes by 23%, reinforcing the rationale for such strategies. Second, adopting a cardio-kidney-metabolic framework represents a major paradigm shift from glucose-centric management to a more holistic, risk-based approach. The substantial cardiovascular benefits observed with SGLT2 inhibitors (55% reduction in heart failure hospitalization) and GLP-1 receptor agonists (35% reduction in major adverse cardiovascular events) highlight the need to align treatment with broader cardiorenal-metabolic risk profiles, rather than focusing exclusively on glycemic control. Third, integrative approaches, including TCM, offer additional therapeutic value. For example, the combination of Huangkui capsules and irbesartan significantly reduced proteinuria (by 262.3 mg/g), suggesting that culturally tailored integrative strategies may benefit select patient populations. However, implementing such approaches requires careful evaluation of evidence quality and vigilant monitoring for herb–drug interactions. Finally, emerging insights into novel disease mechanisms—including ferroptosis, gut microbiota dysbiosis, and epigenetic regulation—provide a foundation for precision medicine. Incorporating biomarkers derived from these pathways may enable personalized therapy selection and dynamic treatment monitoring, accelerating the transition from empirical regimens to mechanism-driven care in DKD.

### 7.2 Research priorities for future investigation

To improve both clinical outcomes and translational progress in DKD, future research should prioritize the following six domains.

#### 7.2.1 Priorities for clinical studies

Large-scale, long-duration randomized controlled trials (RCTs) are urgently needed to evaluate how emerging combination therapies impact hard renal endpoints, including serum creatinine doubling, progression to kidney failure, and renal death. Most existing studies have follow-up durations of 24 weeks to 2.6 years, limiting insight into long-term efficacy and safety. Additionally, head-to-head comparisons—especially regarding the optimal sequencing of SGLT2i, GLP-1 receptor agonists, and non-steroidal MRAs—remain scarce and warrant systematic evaluation.

#### 7.2.2 Personalized treatment approaches

Identifying and validating predictive biomarkers—such as genetic variants, urinary proteomic profiles, and circulating microRNAs—should be a central research priority. Integrating these novel indicators with conventional clinical metrics could support risk-stratified patient classification and facilitate individualized therapeutic decision-making.

#### 7.2.3 Studies of underlying mechanisms

Elucidating the molecular interactions among agents used in combination therapy is essential for optimizing dose selection, minimizing adverse effects, and avoiding mechanistic redundancy. In particular, studies should assess the potential synergistic or antagonistic effects between agents targeting converging biological pathways (e.g., SGLT2i and GLP-1 RAs) to inform rational polypharmacy.

#### 7.2.4 Identification of new treatment targets

Recently identified therapeutic targets—including endothelin receptor antagonists, Nrf2 activators, and ferroptosis inhibitors—represent promising areas for intervention. Notably, isoquercetin has shown efficacy in preclinical models by inhibiting STAT3 and CerS6-mediated cGAS–STING signaling, supporting its progression into early-phase clinical trials.

#### 7.2.5 Generating evidence from clinical practice

The development of large, multi-center registries encompassing diverse populations is crucial to assess the real-world safety and effectiveness of combination therapies. Such databases can also detect rare adverse events and improve the generalizability of clinical trial findings. Special attention should be given to older adults, advanced DKD patients (eGFR < 30 ml/min/1.73 m^2^), and underrepresented ethnic groups.

#### 7.2.6 Economic analysis of health interventions

In resource-constrained settings, comprehensive cost-effectiveness analyses are needed to guide the integration of novel therapies into clinical guidelines and public health policies. These evaluations will also support broader, equitable access to evidence-based precision therapies.

In conclusion, advancing these priority areas will catalyze a paradigm shift in DKD care—from controlling progression to targeting disease mechanisms, and from standardized treatment to personalized, precision-based approaches. This evolution will be essential to achieving sustainable, effective, and equitable care for the growing global population affected by DKD.
